# Supplementary material for: Assessing the role of biomarker feedback in a 12-week community weight management programme among overweight men: A pilot study
Source: PLoS One. 2024 Mar 28;19(3):e0299636. doi: 10.1371/journal.pone.0299636 (PMC10977703; doi:10.1371/journal.pone.0299636)
Supplement: S2 Appendix — (DOCX) [file pone.0299636.s002.docx]

**Participant ID:**

**Treatment Questionnaire Concerning Continued Program Participation**

The following questions relate to your reasons for continuing to participate in the weight-loss

program. Different people have different reasons for continuing in such a program, and we want to know how true each of these reasons is for you. There are two groups of questions. The questions in each group pertain to the sentence that begins that group. The scale is:

1 2 3 4 5 6 7

very true

somewhat true

not at all true

Please circle the number that relates to you on each of the questions in the table.

| 1. **I have remained in treatment because:** | |
| --- | --- |
| 1. I won't like myself very much until I lose weight. | 1 2 3 4 5 6 7 |
| 1. People will like me better when I'm thin. | 1 2 3 4 5 6 7 |
| 1. It feels important to me personally to be thinner. | 1 2 3 4 5 6 7 |
| 1. I really want to make some changes in my life. | 1 2 3 4 5 6 7 |
| 1. I'll feel like a failure if I don't. | 1 2 3 4 5 6 7 |
| 1. People will think I'm a weak person if I don't. | 1 2 3 4 5 6 7 |
| 1. I'll feel very bad about myself if I don't. | 1 2 3 4 5 6 7 |
| 1. Others will be angry at me if I don't. | 1. 2 3 4 5 6 7 |
| 1. **I have been following the procedures of the program because:** | |
| 1. I feel like it's the best way to help myself. | 1 2 3 4 5 6 7 |
| 1. **I plan to lose weight because:** | |
| 1. I'll be ashamed of myself if I don't. | 1 2 3 4 5 6 7 |
| 1. I'll hate myself if I can't get my weight under control. | 1 2 3 4 5 6 7 |
| 1. My friends/family don't like the way I look. | 1 2 3 4 5 6 7 |
| 1. Being overweight makes it hard to do many things. | 1 2 3 4 5 6 7 |
| 1. **I have agreed to follow the procedures of the program because:** | |
| 1. I am worried that I will get in trouble with the staff if I don't follow all the guidelines. | 1 2 3 4 5 6 7 |
| 1. I'll feel guilty if I don't comply with all the procedures. | 1 2 3 4 5 6 7 |
| 1. I want others to see that I am really trying to lose weight. | 1 2 3 4 5 6 7 |
| 1. I believe they will help me solve my problem. | 1 2 3 4 5 6 7 |
| 1. It's important to me that my efforts succeed. | 1 2 3 4 5 6 7 |
