# Supplementary material for: Assessing the role of biomarker feedback in a 12-week community weight management programme among overweight men: A pilot study
Source: PLoS One. 2024 Mar 28;19(3):e0299636. doi: 10.1371/journal.pone.0299636 (PMC10977703; doi:10.1371/journal.pone.0299636)
Supplement: S4 Appendix — (PDF) [file pone.0299636.s004.pdf]

SHORT ACTIVE LIVES QUESTIONNAIRE (NON WEB-BASED ADMINISTRATION)

1) In the past 7 days, have you done a continuous walk lasting at least 10 minutes? Yes/ No

If yes:
a) In the past 7 days, on how many days did you do a walk lasting at least ten minutes? *Please circle*

0 1 2 3 4 5 6 7

b) How much time did you usually spend walking on each day that you did the activity?

_____ hours and _____ minutes per day

c) Was the effort you put into walking usually enough to raise your breathing rate? *Please circle*

Yes No

2) In the past 7 days, have you done a cycle ride? Yes/ No

If yes:
a) In the past 7 days, on how many days did you do a cycle ride? *Please circle*

0 1 2 3 4 5 6 7

b) How much time did you usually spend cycling on each day that you did the activity?

_____ hours and _____ minutes per day

c) Was the effort you put into cycling usually enough to raise your breathing rate? *Please circle*

Yes No

3) In the past 7 days, have you done sport, fitness activity (such as gym or fitness classes), or dance? Yes/ No

If yes:
a) In the past 7 days, on how many days did you do a sport, fitness activity (such as gym or fitness classes), or dance? *Please circle*

0 1 2 3 4 5 6 7

b) How much time did you usually spend doing sport, fitness activities, or dance on each day that you did the activity?

_____ hours and _____ minutes per day

c) Was the effort you put into doing sport, fitness activities, or dance usually enough to raise your breathing rate? *Please circle*

Yes No

SHORT ACTIVE LIVES QUESTIONNAIRE (WEB-BASED ADMINISTRATION)

1) In the past 7 days, have you done any of these activities?

- A continuous walk lasting at least 10 minutes
- A cycle ride
- A sport, fitness activity (such as gym or fitness classes), or dance
- None of these

Ask if ticked yes to walking at Q1:

2) In the past 7 days, on how many days did you do a walk lasting at least ten minutes?

3) How much time did you usually spend walking on each day that you did the activity?

4) Was the effort you put into walking usually enough to raise your breathing rate? Yes/No

Ask if ticked yes to a cycle ride at Q1:

5) In the past 7 days, on how many days did you do a cycle ride?

6) How much time did you usually spend cycling on each day that you did the activity?

7) Was the effort you put into cycling usually enough to raise your breathing rate? Yes/No

Ask if ticked yes to sport or fitness activity at Q1:

8) In the past 7 days, on how many days did you do a sport, fitness activity (such as gym or fitness classes), or dance?

9) How much time did you usually spend doing sport, fitness activities, or dance on each day that you did the activity?

10) Was the effort you put into doing sport, fitness activities, or dance usually enough to raise your breathing rate? Yes/No
